# Supplementary material for: Content-rich biological network constructed by mining PubMed abstracts
Source: BMC Bioinformatics. 2004 Oct 8;5:147. doi: 10.1186/1471-2105-5-147 (PMC528731; doi:10.1186/1471-2105-5-147)
Supplement: Additional File 2 — The original results of the above study (non-essential files are deleted to keep the file size under the limit set by BMC bioinformatics). [file 1471-2105-5-147-S2.bz2 › chilibotAdditionalFile2/dip20/65ID7729427E225/html/TAF12_TBP.html]

 


 **TAF12** and **TBP** 
  
Found 58 abstracts in PubMed, retrieved 20.  
 

 What does Google say? 
 PDF only 
| .edu only 

---

**Interactive relationship** (e.g. stimulation, inhibition, etc)

**Neutral relationship**- The human RNA polymerase II transcription factor B TFIID  [ **TBP** ]  consists of TATA binding protein  [ **TBP** ]   **TBP**  and the  **TBP**  associated factor  [ **TAF12** ]  TAF TAF II 170 and can rapidly redistribute over promoter DNA.  Ref: 11585931 Mol Cell Biol, 2001
- The host factors that are known to regulate  **TBP**  activity include phosphorylation by host kinases and association with different  **TBP**  associated factor  [ **TAF12** ]  TAF subunits.  Ref: 11287600 J Virol, 2001
- Finally, our results suggest that  **TBP**  associated factor  [ **TAF12** ]  components of SAGA are differentially required for  **TBP**  binding to SAGA dependent promoters.  Ref: 12370284 Mol Cell Biol, 2002
- In addition, NuA3 contains the  **TBP**  associated factor  [ **TAF12** ] , yTAF II 30, which is also a component of the TFIID  [ **TBP** ] , TFIIF, and SWI SNF complexes.  Ref: 10817755 Genes Dev, 2000
- The MADF domain directs sequence specific DNA binding to a site consisting of multiple trinucleotide repeats, while the BESS domain directs a variety of protein protein interactions, including interactions with itself, with Dorsal, and with a  **TBP**  associated factor  [ **TAF12** ] .  Ref: 12459265 Gene, 2002
- The TATA box binding activity of transcription factor IID TFIID  [ **TBP** ]  is autoinhibited by the N terminal domain of the Drosophila TATA box binding protein  [ **TBP** ]   **TBP**  associated factor  [ **TAF12** ]  230 yeast  **TBP**  associated factor  [ **TAF12** ]  145 subunit, which binds to the TATA box binding domain of  **TBP**  by mimicking the TATA box structure.  Ref: 10852950 Proc Natl Acad Sci U S A, 2000

**Non-interactive relationship** (e.g. studied together, co-existance, homology, etc.)

- E7 is capable of recognizing a variety of proteins, including RB105, TATA box binding protein  [ **TBP** ]   **TBP** ,  **TBP**  associated factor  [ **TAF12** ]  TAF II 110, E2F, cyclins A and D, and c jun.  Ref: 10881991 J Hum Virol< MedlineTA>J Hum Virol, 2000
- Transcriptional activation by c Jun depends on the TATA binding protein  [ **TBP** ]   **TBP**  associated factor  [ **TAF12** ]  TAF subunits of transcription factor IID TFIID  [ **TBP** ] .  Ref: 11316804 J Biol Chem, 2001
- Our studies indicate that Ii induced B cell maturation involves activation of transcription mediated by the NF kappaB p65 RelA homodimer and requires the B cell enriched coactivator  **TBP**  associated factor  [ **TAF12** ]  II 105.  Ref: 11371575 J Biol Chem, 2001
- Electrophoretic mobility shift assays and DNase I footprinting revealed that acetyl CoA increased the affinity of the general transcription factor TFIID  [ **TBP** ]  for promoter DNA in a  **TBP**  associated factor  [ **TAF12** ]  TAF dependent manner.  Ref: 10688640 Mol Cell Biol, 2000
- It has been demonstrated that the inclusions in dentatorubral pallidoluysian atrophy DRPLA and Machado Joseph disease MJD were immunopositive for several transcription factors such as TATA binding protein  [ **TBP** ]   **TBP** ,  **TBP**  associated factor  [ **TAF12** ]  TAF II 130, Sp1, cAMP responsive element binding protein CREB and CREB binding protein, suggesting that neuronal degeneration in polyglutamine diseases may result from nuclear depletion of transcription factors containing the glutamine rich domain.  Ref: 11211058 Neuropathology, 2000
- The localization of the TATA binding protein  [ **TBP** ]   **TBP**  associated factor  [ **TAF12** ]  II70 TAFII70 in the germinal vesicle GV of newt oocytes was investigated.  Ref: 11768213 Genome, 2001
- Inhibition of host transcription by vesicular stomatitis virus involves a novel mechanism that is independent of phosphorylation of TATA binding protein  [ **TBP** ]   **TBP**  or association of  **TBP**  with  **TBP**  associated factor  [ **TAF12** ]  subunits.  Ref: 11287600 J Virol, 2001
- A composite nuclear export signal in the  **TBP**  associated factor  [ **TAF12** ]  TAFII105.  Ref: 11567023 J Biol Chem, 2001
- TFIID  [ **TBP** ]  and SAGA contain common TATA binding protein  [ **TBP** ]   **TBP**  associated factor  [ **TAF12** ]  TAF II subunits and each complex contains a subunit with histone acetyltransferase activity.  Ref: 10864329 Nature, 2000
- Of these 400, interferon regulatory factor 4 IRF4, cyclin B2,  **TBP**  associated factor  [ **TAF12** ] , eukaryotic elongation factor and pim 2 were up regulated more than 3.5 fold.  Ref: 11790884 Dis Markers, 2001
- Interestingly, a set of genes affected in the taf1 DeltaTAND mutant is similarly affected in the  **taf12**  HFD mutants but not in the nsl mutants of  **TBP** .  Ref: 12582246 Nucleic Acids Res, 2003
- The next gene in this map, located distal to SS18, was found to be the  **TBP**  associated factor  [ **TAF12** ]  TAFII 105 TAF2C2.  Ref: 11435705 Cytogenet Cell Genet, 2001
- In vitro, holo TFIID  [ **TBP** ]  and  **TBP**  associated factor  [ **TAF12** ]  250 TAF II 250 phosphorylated TFIIA on the beta subunit.  Ref: 11278496 J Biol Chem, 2001
- Mutations in the histone fold domain of the  **TAF12**  gene show synthetic lethality with the TAF1 gene lacking the TAF N terminal domain TAND by different mechanisms from those in the SPT15 gene encoding the TATA box binding protein  [ **TBP** ]   **TBP** .  Ref: 12582246 Nucleic Acids Res, 2003
- Requirement of tissue selective  **TBP**  associated factor  [ **TAF12** ]  TAFII105 in ovarian development.  Ref: 11557891 Science, 2001
